# How feasible is it to mobilize $31 billion a year for pandemic preparedness and response? An economic growth modelling analysis

**DOI:** 10.1186/s12992-024-01058-4

**Published:** 2024-07-19

**Authors:** Minahil Shahid, Marco Schäferhoff, Garrett Brown, Gavin Yamey

**Affiliations:** 1 Center for Policy Impact in Global Health, Duke University, Durham, NC USA; 2 Duke Global Health Institute, Duke University, Durham, NC USA; 3 Open Consultants, Berlin, Germany; 4 School of Politics and International Studies, University of Leeds, Leeds, UK

**Keywords:** Pandemic preparedness and response, COVID-19, Low- and middle-income countries, Health financing

## Abstract

**Background:**

Covid-19 has reinforced health and economic cases for investing in pandemic preparedness and response (PPR). The World Bank and World Health Organization (WHO) propose that low- and middle-income governments and donor countries should invest $31.1 billion each year for PPR. We analyse, based on the projected economic growth of countries between 2022 and 2027, how likely it is that low- and middle-income country governments and donors can mobilize the estimated funding.

**Methods:**

We modelled trends in economic growth to project domestic health spending by low- and middle-income governments and official development assistance (ODA) by donors for years 2022 to 2027. We modelled two scenarios for countries and donors – a constant and an optimistic scenario. Under the constant scenario we assume that countries and donors continue to dedicate the same proportion of their health spending and ODA as a share of gross domestic product (GDP) and gross national income (GNI), respectively, as they did during baseline (the latest year for which data are available). In the optimistic scenario, we assume a yearly increase of 2.5% in health spending as a share of GDP for countries and ODA as a share of GNI for donors.

**Findings:**

Our analysis shows that low-income countries would need to invest on average 37%, lower-middle income countries 9%, and upper-middle income countries 1%, of their total health spending on PPR each year under the constant scenario to meet the World Bank WHO targets. Donors would need to allocate on average 8% of their total ODA across all sectors to PPR each year to meet their target.

**Conclusions:**

The World Bank WHO targets for PPR will not be met unless low- and middle-income governments and donors spend a much higher share of their funding on PPR. Even under optimistic growth scenarios, low-income and lower-middle income countries will require increased support from global health donors. The donor target cannot be met using the yearly increase in ODA under any scenario. If the country and donor targets are not met, the highest-impact health security measures need to be prioritized for funding. Alternative sources of PPR financing could include global taxation (e.g., on financial transactions, carbon, or airline flights), cancelling debt, and addressing illicit financial flows. There is also a need for continued work on estimating current PPR costs and funding requirements in order to arrive at more enduring and reliable estimates.

**Supplementary Information:**

The online version contains supplementary material available at 10.1186/s12992-024-01058-4.

## Introduction

The covid-19 pandemic has had devastating health and social consequences, causing death, disability (e.g., from Long Covid), and orphanhood. The World Health Organization (WHO) estimates over 7 million deaths resulting from covid-19 as of January 2024 [1], while the economic losses will be close to US$ 13.8 trillion from 2020 to 2024 according to the International Monetary Fund [2]. Even before the pandemic, major gaps had been identified in the global health security architecture [3]. Covid-19 has reinforced the health and economic case for investing in pandemic preparedness and response (PPR) [4]. Such investments are argued to help prevent, detect, and contain disease outbreaks, thereby reducing the broader social and economic costs of a pandemic [4, 5].

How much would it cost to establish a global PPR system that is fully fit-for-purpose? Despite ongoing dialogue, there is currently no consistently applied approach to calculating global PPR resource requirements [6]. Previous estimates have ranged from US$ 1.6 billion to US$ 43 billion per year, depending on the costing methodology used, preparedness activities considered, and countries included in the analysis [7].

The World Bank and the World Health Organization (WHO) recently provided a new estimate of the annual PPR financing needs in a report conducted for the G20 Joint Finance and Health Task Force [5]. The international community is now coalescing around these new figures. The World Bank and WHO estimate that low- and middle-income country governments and donors need to invest US$ 31.1 billion annually in PPR, of which US$ 26.4 billion needs to be invested at the country level and US$ 4.7 billion at the international level [5, 8]. The report also acknowledges that low- and lower-middle income countries are unlikely to meet their national PPR financing requirements, estimating that there is an overall annual funding gap of US$ 10.5 billion at global and country levels [5].

A critical question to answer is: assuming the figure is correct, how feasible is it to achieve this annual “price tag” of US$ 31.1 billion? We therefore set out to address this question by analysing, based on the economic growth that the International Monetary Fund projects for years 2022 to 2027 [9], how likely it is that low- and middle-income countries and donors will mobilize the estimated funding for PPR.

Considering various scenarios, we addressed two questions. First, how realistic is it for low- and middle-income governments to reach the annual country level PPR finance target of US$ 26.4 billion from growth in domestic health spending? Second, how feasible is it for donors to support low- and middle-income countries to reach this US$ 26.4 billion target, while at the same time financing global (international-level) PPR needs? In addition, we challenge the US$ 10.5 billion annual funding gap identified by the World Bank/WHO, suggesting that it is based on poor assumptions, and that the funding gap is actually closer to US$ 15.5 billion. Table 1 summarizes how we defined low- and middle-income countries and donors and Table 2 summarizes the World Bank/WHO’s PPR financing report estimates.

**Table 1:** Definition of low- and middle-income countries and donors

We conducted separate analyses for low- and middle-income countries and donors:
• **Low- and middle-income countries.** We used the World Bank’s classification of countries by income group [10] to identify and conduct assessment for: low-income countries (LICs), lower-middle-income countries (LMICs), and upper-middle-income countries (UMICs). In total, we included 115 countries in our analysis (Appendix 1). Seventeen countries were excluded due to lack of data.
• **Donor countries**. We identified donor countries using the Organisation for Economic Co-operation and Development (OECD) Disbursements and Commitments of Official and Private Flows Statistics Database [11, 12]. Our analysis included a total of 43 donor countries—of which 29 are members of the OECD Development Assistance Committee (DAC) [13] and 14 are non-DAC donors (Appendix 2). Five donor nations (Azerbaijan, Chinese Taipei, Kazakhstan, Kuwait, and Liechtenstein) were excluded due to lack of data.

**Table 2:** Summary of WHO and World Bank’s 2022 PPR financing estimates

**Country-level PPR financing:**
• Low- and middle-income countries’ PPR financing needs are estimated at US$ 26.4 billion per year ($2.7 billion for low-income, $13.5 billion for lower-middle income, and $10.2 billion for upper-middle income countries).
• The report makes two assumptions: 1) National governments already invest 3% of their healthcare spending on PPR; 2) A significant amount of international funding (which includes $14.1 billion in domestic resources and multilateral development bank (MDB) financing, $1.0 billion in bilateral aid, $0.6 billion in multilateral aid, $1.4 billion in targeted pooled mechanisms, $1.6 billion in private sector, and $0.2 billion in philanthropy) is already supporting national level PPR requirements. 100% of low-income country, 60% of lower-middle income country, and 20% of upper-middle income country PPR needs are already supported by this international funding.
• Existing international funding amounts to $18.9 billion in total and reduces the country PPR gap to $7.5 billion, of which $7.0 billion is identified as additional international financing need.
**Global level PPR financing:**
• Global (including regional) PPR financing needs are estimated at $4.7 billion.
• It is assumed that existing institutions and funding mechanisms have capacity to contribute 25% of the need, resulting in a gap of $3.5 billion in international financing.
**Consolidated PPR financing gap:**
• PPR financing gap is the sum of $7 billion at the country-level and $3.5 billion at the global level, or $10.5 billion in total.

## Methods

Our study did not obtain an ethics approval as it does not involve human subjects and only uses publicly available national and international financial data and published secondary resources.

### Low- and middle-income countries’ analysis

Using low- and middle-income country gross domestic product (GDP) data from the World Bank [14] for the year 2021, we projected the GDP for each country from 2022 to 2027 using the annual percentage change in GDP data from the International Monetary Fund’s (IMF) World Economic Outlook (WEO) database [9]. We used real GDP to account for the effects of inflation across all analyses. After calculating the projected economic growth (in constant 2020 US$) for low- and middle-income countries, we used the ‘Domestic General Government Health Expenditure (GGHE-D) (also referred to as ‘domestic health spending’) as a percent of GDP’ data from the WHO Global Health Expenditure database [15], and multiplied these values with the projected GDP data from the WEO dataset to get the projected domestic health spending by low- and middle-income countries for the years 2022 to 2027. For the projected years, we calculated two different scenarios:

1. Constant Scenario: We assumed that the share of government health expenditures out of GDP remained constant (i.e. we assumed that the latest available ‘GGHE-D as a percent of GDP’ data, which are currently for the year 2020, apply to all years from 2022 to 2027). In other words, in this constant scenario, low- and middle-income countries continue to spend the same percentage of their GDP on domestic health spending between the years 2022 to 2027 as they did in 2020.
2. Scale-up Scenario: We increased the ‘GGHE-D as a percent of GDP’ ratio by 2.5 percent each year up until 2027. This is a more optimistic scenario, one in which low- and middle-income countries recognize the need to marginally increase the percentage of their GDP spent on health year on year.

For both scenarios, we projected the trend in GGHE-D for each income group and calculated what share of GGHE-D would be required to meet the annual US$ 26.4 billion PPR target by income group (Table 3 shows the target cost breakdown by income group). In addition, we examined the yearly increment in domestic health spending under the two scenarios to estimate what proportion of the increment would be needed to meet the PPR target. Our analysis focuses on the anticipated growth in GGHE-D, and it does not examine any redistribution of domestic health spending from other priority areas such as infectious disease control or maternal and child health. We used GDP data from the World Bank [14] and calculations were performed in 2020 US$.

**Table 3:** Estimated national-level annual PPR target by income group

**Income Group**	**Cost (Billion US$)**	**Cost (%)**
LIC Target	2.7	10.2%
LMIC Target	13.5	51.1%
UMIC Target	10.2	38.6%
**Total**	**26.4**	**100.0%**

### Donor analysis

We estimated the increase or decrease in official development assistance (ODA) that donor countries are projected to give between the years 2022 and 2027 using projected changes to their economic growth (i.e., changes in their gross national income [GNI]). We used GNI rather than GDP due to limitations in data availability; there is a relatively small difference in values between the two indicators (i.e., using GNI or GDP would give similar results).

We used the annual percentage change in GDP data for years 2022-2027 from the IMF’s WEO database. We multiplied this percentage change with the Gross National Income (GNI) data for donor countries for the year 2021 from the OECD DAC1 [11] database to calculate the projected economic growth for DAC and non-DAC donors for the years 2022 to 2027. The OECD DAC1 database provides historical data on disbursements and commitments of official and private flows from members of the Development Assistance Committee (DAC), multilateral organisations and other donors.

We then used the ‘Official Development Assistance (ODA) as a percent of GNI’ data from the OECD DAC1 database to calculate the projected ODA flows by donors for the years 2022 to 2027. We created two scenarios (similar to the country-level analyses) described below:

1. Constant Scenario: Keeping the ODA/GNI ratio constant, we assumed that donors continue to give the same percent of their GNI to ODA between 2022 to 2027 as they did in 2021; in this scenario, any increase or decrease in ODA is solely driven by changes in donors’ GNI data;
2. Scale-Up Scenario: We assume that there is a yearly 2.5 percent increase in the 2021 ODA to GNI ratio, compounding to a total increase of 15 percent by 2027.

To capture different scenarios, we varied the proportion of GNI that donor nations might give to ODA between 2022-27 using the 2021 ODA/GNI ratio as the baseline (the latest year for which ODA/GNI ratio data were available). A pessimistic scenario with a decreasing ODA-to-GNI ratio can potentially stem from budgetary pressures and ODA cuts by certain donor countries. In contrast, an optimistic view would suggest that, for example, given the economic and social losses caused by covid-19, donors find value in investing a greater proportion of their GNI towards ODA, leading to an overall increase in ODA availability. In line with our country-level GGHE-D analysis, we projected ODA growth from 2022-2027 under two scenarios – a constant and a scale-up scenario.

In our analysis, we assume that donors would cover the entire global-level PPR investment of US$ 4.7 billion, and also provide 100% of the annual PPR funding for LICs (US$ 2.7 billion per year) and 60% for LMICs (US$ 8.1 billion per year). The assumptions on the share of funding covered by donors reflects those in the World Bank/WHO costing study. In total, we assume donors would need to provide an annual amount of US$ 15.5 billion (rationale further discussed in the Discussion section).

The Institute for Health Metrics and Evaluation (IHME), in their 2023 report on the future of financing global health, [16] forecasts two scenarios between 2019 and 2030 – one where development partners continue to prioritise health, delivering US $50.6 billion in development assistance for health by 2030 (an increase of 4.9%), and a scenario where development partners prioritise other sectors, reducing assistance to as low as US $36.7 billion (a decrease by 23.9%). In the latter scenario, the reductions in assistance would negatively affect PPR investments even if PPR was still prioritized over other health needs. In that case, and in relation to our research question, it is near certain that PPR estimates would not be reached by ODA assistance nor by country-level investments. As a result, we focus our research on the assumptions that levels remain constant or are slightly scaled-up, to determine whether even in better-case scenarios it would be possible to meet the estimated costs of PPR. If it is still not feasible, it bears several important implications for ongoing discussions about PPR financing.

## Results

### Low- and middle-income countries analysis results

Our analysis shows that LICs, under the constant scenario, would have to invest 42.2% of their total annual GGHE-D to reach their PPR target in 2022, while in 2027 they would still need to spend 31.2% of their GGHE-D on PPR (Table 4). Under the scale-up scenario, LICs would have to invest 26.9% of their GGHE-D in 2027 to meet the PPR target (Table 5). The increment in funding under both constant and scale-up scenarios is insufficient for reaching the PPR target for LICs.

**Table 4:** Projected growth in domestic health spending by low- and middle-income country governments under the constant scenario

**Indicator**	**2022**	**2023**	**2024**	**2025**	**2026**	**2027**	**Mean**
GGHE-D^a^ LIC (Billion US$)	6.4	6.7	7.2	7.6	8.1	8.7	7.4
GGHE-D LMIC (Billion US$)	129.6	132.1	139.5	147.2	155.2	163.5	144.5
GGHE-D UMIC (Billion US$)	976.4	1,011.9	1,052.4	1,092.6	1,134.2	1,176.4	1,074.0
**Total GGHE-D (Billion US$)**	**1,112.4**	**1,150.7**	**1,199.0**	**1,247.5**	**1,297.5**	**1,348.6**	**1,225.9**
PPR as a % of GGHE-D (LIC)^b^	42.2%	40.1%	37.7%	35.4%	33.2%	31.2%	36.6%
PPR as a % of GGHE-D (LMIC)^c^	10.4%	10.2%	9.7%	9.2%	8.7%	8.3%	9.4%
PPR as a % of GGHE-D (UMIC)^d^	1.0%	1.0%	1.0%	0.9%	0.9%	0.9%	1.0%
**Total PPR as a % of Total GGHE-D**	**2.4%**	**2.3%**	**2.2%**	**2.1%**	**2.0%**	**2.0%**	**2.2%**
Increment^e^ (LIC) (Billion US$)		0.3	0.4	0.5	0.5	0.5	0.5
Increment (LMIC) (Billion US$)		2.4	7.4	7.8	8.0	8.3	6.8
Increment (UMIC) (Billion US$)		35.6	40.4	40.3	41.5	42.3	40.0
**Total Increment (Billion US$)**		**38.4**	**48.2**	**48.5**	**50.0**	**51.1**	**47.2**
PPR as a % of Increment (LIC)^f^		800.2%	643.4%	586.4%	535.7%	509.7%	615.1%
PPR as a % of Increment (LMIC)^g^		553.4%	182.7%	173.6%	169.3%	162.5%	248.3%
PPR as a % of Increment (UMIC)^h^		28.7%	25.2%	25.3%	24.6%	24.1%	25.6%
**Total PPR as a % of Total Increment**		**68.8%**	**54.7%**	**54.4%**	**52.8%**	**51.7%**	**56.5%**

^a^GGHE-D (domestic general government health expenditure) was retrieved from the WHO Global Health Expenditure database [15]

^b^Proportion of GGHE-D that would need to be directed towards PPR to meet the LIC target (targets available in Table 3)

^c^Proportion of GGHE-D that would need to be directed towards PPR to meet the LMIC target

^d^Proportion of GGHE-D that would need to be directed towards PPR to meet the UMIC target

^e^Increment is calculated by subtracting the previous year’s GGHE-D from the current year’s value

^f^Proportion of increment that would need to be directed towards PPR to meet the LIC target

^g^Proportion of increment that would need to be directed towards PPR to meet the LMIC target

^h^Proportion of increment that would need to be directed towards PPR to meet the UMIC target

**Table 5:** Projected growth in domestic health spending by low- and middle-income country governments under the scale-up scenario

**Indicator**	**2022**	**2023**	**2024**	**2025**	**2026**	**2027**	**Mean**
GGHE-D^a^ LIC (Billion US$)	6.6	7.1	7.7	8.4	9.2	10.0	8.2
GGHE-D LMIC (Billion US$)	132.9	138.7	150.2	162.5	175.6	189.6	158.3
GGHE-D UMIC (Billion US$)	1,000.8	1,063.2	1,133.3	1,206.1	1,283.2	1,364.3	1,175.1
**GGHE-D Total (Billion US$)**	**1,140.2**	**1,209.0**	**1,291.2**	**1,377.0**	**1,468.0**	**1,564.0**	**1,341.5**
PPR as a % of GGHE-D (LIC)^b^	41.2%	38.1%	35.0%	32.1%	29.4%	26.9%	33.8%
PPR as a % of GGHE-D (LMIC)^c^	10.2%	9.7%	9.0%	8.3%	7.7%	7.1%	8.7%
PPR as a % of GGHE-D (UMIC)^d^	1.0%	1.0%	0.9%	0.8%	0.8%	0.7%	0.9%
**Total PPR as a % of Total GGHE-D**	**2.3%**	**2.2%**	**2.0%**	**1.9%**	**1.8%**	**1.7%**	**2.0%**
Increment^e^ (LIC) (Billion US$)		0.5	0.6	0.7	0.8	0.8	0.7
Increment (LMIC) (Billion US$)		5.9	11.4	12.3	13.1	14.0	11.4
Increment (UMIC) (Billion US$)		62.4	70.1	72.8	77.1	81.1	72.7
**Total Increment (Billion US$)**		**68.8**	**82.2**	**85.8**	**91.0**	**96.0**	**84.8**
PPR as a % of Increment (LIC)^f^		520.7%	429.3%	385.2%	346.0%	319.9%	400.2%
PPR as a % of Increment (LMIC)^g^		229.4%	118.2%	109.4%	103.2%	96.3%	131.3%
PPR as a % of Increment (UMIC)^h^		16.3%	14.5%	14.0%	13.2%	12.6%	14.1%
**Total PPR as a % of Total Increment**		**38.4%**	**32.1%**	**30.8%**	**29.0%**	**27.5%**	**31.6%**

^a^GGHE-D (domestic general government health expenditure) was retrieved from the WHO Global Health Expenditure database [15]

^b^Proportion of GGHE-D that would need to be directed towards PPR to meet the LIC target (targets available in Table 3)

^c^Proportion of GGHE-D that would need to be directed towards PPR to meet the LMIC target

^d^Proportion of GGHE-D that would need to be directed towards PPR to meet the UMIC target

^e^Increment is calculated by subtracting the previous year’s GGHE-D from the current year’s value

^f^Proportion of increment that would need to be directed towards PPR to meet the LIC target

^g^Proportion of increment that would need to be directed towards PPR to meet the LMIC target

^h^Proportion of increment that would need to be directed towards PPR to meet the UMIC target

For LMICs, the percentage of GGHE-D needed to meet their PPR target drops from 10.4% in 2022 to 8.3% in 2027 under the constant scenario and to 7.1% in the scale-up scenario. The increment in projected funding is also insufficient for LMICs to meet their PPR costs.

For UMICs, the annual PPR costs account for 1.0% of their total annual GGHE-D in the constant scenario, and drop to 0.7% in 2027 under the scale-up scenario. UMICs, unlike LICs and LMICs, would be able to cover PPR costs using a substantial share of their incremental funding – 25.6% on average under the constant scenario and 14.1% on average under the scale-up scenario.

A limitation of our analysis stems from the composition of the GGHE-D indicator in the WHO Global Health Expenditure database. The indicator does not account for capital expenditures while calculating domestic health spending [17], which leads to an overall underestimation in projected GGHE-D. We therefore conducted a sensitivity analysis (see Appendix 3), and included capital expenditures (reported separately in the WHO database) in our analysis.

### Donor analysis results

Our analysis shows that the US$ 15.5 billion annual PPR target for donor government spending would not fully be met under any scenario, even if the entire yearly increase in ODA is used for PPR. Even under the scale-up scenario of a 15% linear increase in the ODA/GNI ratio, and even if the entire yearly increase in ODA over the six years is directed to PPR, it would only cover an annualised average of 61% of the US$ 15.5 billion annual PPR requirement (see Table 6), and 90% if the target was brought down to US$ 10.5 billion.

**Table 6:** Projected growth in official development assistance by donors under the constant and scale-up scenarios

	**2022**	**2023**	**2024**	**2025**	**2026**	**2027**	**Mean**
**Constant Scenario**							
ODA (Billion US$)	186.2	190.5	193.7	197.0	200.2	203.3	195.1
PPR as a % of ODA^a^	8.3%	8.1%	8.0%	7.9%	7.7%	7.6%	7.9%
Increment (Billion US$)	7.4	4.2	3.2	3.3	3.2	3.1	4.1
Increment as a % of PPR^b^	48.0%	27.4%	20.9%	21.1%	20.5%	19.9%	26.3%
**Scale-Up Scenario**							
ODA (Billion US$)	190.9	200.1	208.6	217.5	226.5	235.7	213.2
PPR as a % of ODA^a^	8.1%	7.7%	7.4%	7.1%	6.8%	6.6%	7.3%
Increment (Billion US$)	12.1	9.2	8.5	8.8	9.0	9.2	9.5
Increment as a % of PPR^b^	78.0%	59.6%	54.8%	57.0%	58.2%	59.6%	61.2%

^a^Proportion of ODA that would need to be directed towards PPR to meet the US$ 15.5 billion target each year

^b^Proportion of annual US$ 15.5 billion target that would be met if the entire increment was directed at PPR

## Discussion

Our analysis for the constant scenarios shows that LICs would need to invest on average 37%, LMICs 9%, and UMICs 1%, of their total health spending on PPR each year to meet the World Bank WHO targets. Donors would need to allocate on average 8% of their total ODA across all sectors to PPR each year to meet their target.

Based on these projections, we believe it is not feasible for low- and lower-middle-income governments to reach their annual PPR funding targets from domestic spending alone. Even under the optimistic scenario, LICs would still have to allocate, on average, 34% of their total GGHE-D between 2022 and 2027 to PPR, making the target untenable. The largest PPR costs relate to LMICs – a diverse group of countries with variable abilities to pay their own PPR needs. Our analysis shows that LMICs would need to spend on average 7-10% of their GGHE-D on PPR between 2022 and 2027.

In terms of the increment, under the constant scenario, the increment resulting from economic growth is insufficient for LMICs to meet their PPR requirements (the increment for LMICs, on average from 2022 to 2027, is US$ 6.8 billion, while the requirement is US$ 13.5 billion per year). One potential implication is that LMICs would need to reduce their health spending on other priorities to meet the target.

In addition, many LMICs will lose support from the Global Fund, Gavi, the Vaccine Alliance, and other donors in the coming years and will need to increase their domestic spending on priorities such as HIV, tuberculosis, malaria, and vaccination programs.[18] Their likely economic growth will allow them to increase their health spending, but given competing priorities it is unrealistic to suggest that more than the entire growth in projected health funding will be directed towards PPR because that would imply a reallocation from other health areas. Moreover, a recent IHME report [16] found that debt servicing on loans taken during covid-19 has been exacerbated by high interest rates and is estimated to have reduced health budgets, especially for low-income countries, which saw a decrease of 8.6% on average between 2020 and 2023. If these calculations are right, then it further increases the unlikelihood of low- and middle-income countries to meet their PPR targets.

According to our analysis, UMICs are deemed able to finance their own PPR target, and only need to dedicate on average 1% of their GGHE-D, or 14-26% of their increment, to PPR. Thus, it is clear that donors would only need to support LICs and LMICs. Given the constraints of these country groups to self-finance their PPR needs, it is important to be realistic and transparent about the amount of additional donor funding required. The World Bank/WHO costing study argues that the annual priority need at country-level is US$ 26.4 billion, of which US$ 16.2 billion or 61.3% would fall on LICs and LMICs, while the remainder is for UMICs, who, according to the World Bank, can cover a majority of those costs themselves. Because the World Bank/WHO study assumes that donors and other international financing sources already cover 100%, 60% and 20% of the LIC, LMIC and UMIC costs respectively, the annual funding gap at country level is reduced from $26.4 billion to $7.0 billion.

We challenge the assumption that such a significant amount of funding is currently being directed towards PPR. The World Bank/WHO report assumes that donors already provide $1.0 billion in bilateral aid and $0.6 billion in multilateral aid. However, according to a recent Lancet study, in 2021 only $786.6 million in development assistance went to PPR, with this amount being on the higher end due to the pandemic [19]. There was a significant increase in health ODA in 2020 and 2021 (from US$ 22.2 billion in 2019 to US$ 29.2 billion in 2020 and US$ 34.0 billion in 2021), and much of this increase can be attributed to the covid-19 response, especially donor funding for covid-19 vaccines [20]. In previous years, donors have invested very little in PPR [21]. In addition, there is evidence that existing ODA and national level resources for health have shifted to covid-19 and PPR activities, signalling a reallocation of scarce resources which can threaten existing Universal Health Coverage (UHC) vulnerabilities [22].

The World Bank/WHO report also assumes that $14.1 billion in funding is already available via domestic and MDB financing, including low- and middle-income countries which are investing 3% of their government health spending on PPR. There is currently no widely accepted measurement of domestic spending on PPR, but 3% might be an overestimation as reports state the figure varies between 1% and 3% [5]. A recent IHME report also noted that due to higher debt and interest rates since covid-19, low-income country health spending has actually *decreased* by $1 per person between 2020 and 2023, [16] rendering a higher health spending estimate untenable. The World Bank/WHO report further assumes that 25% of the global and regional PPR needs are already covered via existing institutions. However, pooled funds and MDB financing including the Pandemic Fund and agencies like the Global Fund, GAVI, the Vaccine Alliance, Coalition for Epidemic Preparedness Innovations (CEPI), also included in the report’s list of current contributors, are struggling to meet their current financing targets [23–25]. The World Bank’s Pandemic Fund has struggled to mobilise the necessary funding to cover only three core capacities associated with PPR in low-income countries (surveillance, diagnostics, and human resource for those activities), and was eight-times oversubscribed in its first round of funding with a gap of over US$ 2.1 billion [26]. As a result, the assumptions of the World Bank/WHO study about existing donor funding appear to be unrealistic.

We argue that the annual donor funding needed amounts to US$15.5 billion – US$ 4.7 billion for global and regional PPR (assuming that 25% of this global PPR requirement is not already covered by international funding), US$ 2.7 billion per year for LICs (total LIC costs for PPR, assuming that 100% of LIC costs are not already covered by domestic and international financing) and US$ 8.1 billion per year for LMICs (60% of the total LMIC costs, assuming that 60% of LMIC costs are not already covered by domestic and international financing). The G20 High Level Independent Panel on Pandemic Preparedness and Response (HLIP) in a separate study estimating the cost of PPR also concluded that international financing needs to increase by an additional US$15 billion per year for the next 5 years [27]. This is in line with our understanding that calculating the annual gap requires more realistic data and assumptions on donor spending for PPR during non-pandemic times as well as a recognition that the annual funding gap is higher than US$ 10.5 billion.

Our donor analysis is also using 2021 as the baseline year for the ODA/GNI ratio for years 2022 to 2017 under the constant or more conservative scenario, despite the fact that donors were spending a higher share of their GDP on health due to the pandemic in 2021. With this overestimation, and more ODA being recently redirected to humanitarian crises like Ukraine and Gaza [28], our assumption of a bigger donor target that covers 100% of LIC and 60% of LMIC costs should hold.

How feasible is it to mobilize US$15.5 billion in donor funding for PPR every year through 2027? Donors would need to allocate 7-8% of their total ODA – across all sectors - to PPR between 2022 and 2027. In terms of the increment in total ODA, on average, the increment could cover 26% and 61% of the PPR requirement under the constant and scale-up scenarios, respectively. In other words, even if the entire increase in ODA increment is spent on PPR, which is unrealistic in itself, donors would not be able to meet this funding without sufficient increases in the percentage of ODA allocated to PPR via new funding. Ideally, this would not merely be a redistribution from other ODA commitments.

How might the global health community respond to these projections? One approach is to simply accept these projections and design plans for how to efficiently spend whatever financing does get mobilized by identifying and prioritising the highest value for money and PPR impact measures. Another, and potentially complementary, approach would be for donors to increase their funding beyond the 2.5% ODA/GNI ratio as a PPR investment strategy against the type of public health and economic risk experienced during covid-19. Here, there are arguments to be made from a benefit/cost perspective that could make ODA/GNI increases more palatable to donors and their constituents.

In addition, both the G20 HLIP and World Bank/WHO studies advocate for wider instruments and funding sources beyond ODA to be marshalled to meet PPR shortfalls, including the greater use of innovative financing, pooled funds, increased private sector partnerships, additionality, and the identification of new multisectoral sources and funders. This strategy to move away from an overreliance on ODA has also been included in the revised and approved International Health Regulations (IHRs), where a new Coordinating Financial Mechanism has been outlined [29, 30]. In theory, such a coordination mechanism could harmonize existing funding, provide the means for continuous and more reliable PPR gap analysis for both IHR compliance as well as specific PPR activities, which would still produce common goods and might instill some donor confidence. However, in terms of mobilizing enough funding, it is easier said than done, since recent initiatives such as the Pandemic Fund have so far remained largely business as usual and reliant on the same funding sources and instruments (aside from the inclusion of The Wellcome Trust). As a result, any new PPR or IHR coordination mechanism would face significant challenges to instill a major step-change away from how global health has traditionally been financed. Given that negotiations on the Pandemic Agreement have now been extended until May 2025, and that the details of the IHR Coordinating Financial Mechanism are very much ‘to be determined’, it is hard to foresee what impacts on PPR financing they will have.

Other alternative sources of financing outside the usual health related sources can also be explored to address PPR financing needs (e.g., from national security or defence budgets) [31]. For example, there is growing interest in levying a global tax on financial transactions, carbon, or airline flights to help fund PPR [32]. Debt cancellation must also be on the table. Public debt in low- and middle-income countries increased from 58% to 65% of GDP from 2019 to 2021 [33]. The cost of borrowing for low-income countries has also increased compared to pre-pandemic levels and is projected to continue increasing as global interest rates rise [33]. If the G20 and financial institutions had cancelled all external debt payments due in 2020 and 2021 by the 76 poorest countries, it would have liberated US$ 300 billion [34].

Addressing illicit financial flows (IFFs) and global tax abuses that continue to trickle wealth from low- and middle-income countries into higher-income nations could also help redirect resources from illicit channels into more productive ones such as investing in PPR. Eastern and Southern Africa lost US$ 7.6 billion in tax revenue in 2017, equivalent to 1.6% of the region’s GDP, due to only two sources of IFFs (base erosion and profit shifting to tax havens) [35]. Addressing IFFs is important, since countries with high IFFs are reported to spend 25% less on health compared to countries with low IFFs [36]. Measures to tackle IFFs can strengthen financial systems and also free resources for public health purposes.

Reducing the cost of PPR itself is also desirable and would require strong measures including reducing constraints on intellectual property (IP) to allow equitable global access to safe and affordable medical countermeasures (MCMs). During covid-19, pharmaceutical companies partnered with certain high-income countries to hinder IP waiver negotiations, stalling progress towards equitable access to covid-19 vaccines [37, 38]. Solutions to these issues could help to reduce PPR costs as well as lower resource requirements.

## Conclusion

Assuming that the US$ 31.1 billion estimate is correct, the World Bank WHO targets for PPR will not be met unless low- and middle-income governments and donors spend a much higher share of their funding on PPR. One implication of a continued inability to mobilise new funding from donors could be that resources are shifted from existing country-level health budgets, which will disproportionately compromise countries that already struggle to finance their health systems and programmes such as UHC. This would represent a significant opportunity cost with potentially high negative health outcomes. Given the findings presented in this article, even under optimistic growth scenarios, low-income and lower-middle income countries will require increased support from global health donors. If the country and donor targets are not met, the highest-impact health security measures need to be prioritized for funding. Alternative sources of PPR financing could include global taxation (e.g., on financial transactions, carbon, or airline flights), cancelling debt, and addressing illicit financial flows. There is also a clear need for continued work on estimating current PPR costs and funding requirements in order to arrive at more enduring and reliable estimates.

### Supplementary Information

Supplementary Material 1.

Supplementary Material 2.

Supplementary Material 3.

## Data Availability

We used publicly available datasets for our analysis. These include: International Monetary Fund’s World Economic Outlook database; Organisation for Economic Co-operation and Development (OECD) Disbursements and Commitments of Official and Private Flows Statistics Database (DAC1, CRS); World Health Organization's Global Health Expenditure Database; World Bank's World Development Indicators. Our data analysis will be made available on request.
