# Supplementary material for: How feasible is it to mobilize $31 billion a year for pandemic preparedness and response? An economic growth modelling analysis
Source: Global Health. 2024 Jul 19;20:54. doi: 10.1186/s12992-024-01058-4 (PMC11264850; doi:10.1186/s12992-024-01058-4)
Supplement: Supplementary file 2 — Supplementary Material 2. [file 12992_2024_1058_MOESM2_ESM.docx]

**Appendix 2**

Countries included in the analysis of donor government spending on ODA:

*DAC donors included:*

| Australia | Korea |
| --- | --- |
| Austria | Luxembourg |
| Belgium | Netherlands |
| Canada | New Zealand |
| Czech Republic | Norway |
| Denmark | Poland |
| Finland | Portugal |
| France | Slovak Republic |
| Germany | Slovenia |
| Greece | Spain |
| Hungary | Sweden |
| Iceland | Switzerland |
| Ireland | United Kingdom |
| Italy | United States |
| Japan |  |

*Non-DAC donors included:*

| Bulgaria |
| --- |
| Croatia |
| Cyprus |
| Estonia |
| Israel |
| Latvia |
| Lithuania |
| Malta |
| Qatar |
| Romania |
| Saudi Arabia |
| Thailand |
| Turkey |
| United Arab Emirates |

Countries not included in the donor analysis:

*Non-DAC donors not included:*

| Azerbaijan |
| --- |
| Chinese Taipei |
| Kazakhstan |
| Kuwait |
| Liechtenstein |
